# Proteogenomics Integrating Reveal a Complex Network, Alternative Splicing, Hub Genes Regulating Heart Maturation

**DOI:** 10.3390/genes13020250

**Published:** 2022-01-28

**Authors:** Huijun Yang, Weijing Liu, Shen Song, Lina Bai, Yu Nie, Yongping Bai, Guogang Zhang

**Affiliations:** 1 Department of Cardiovascular Medicine, Xiangya Hospital, Central South University, Changsha 410008, China; y_huijun@126.com; 2 State Key Laboratory of Cardiovascular Disease, Fuwai Hospital, National Center for Cardiovascular Disease, Chinese Academy of Medical Sciences and Peking Union Medical College, Beijing 100037, China; liuwjsx001@163.com (W.L.); songs_193@163.com (S.S.); bailina0520@163.com (L.B.); nieyuniverse@126.com (Y.N.); 3 Department of Geriatric Medicine, Xiangya Hospital, National Clinical Research Center for Geriatric Disorders, Central South University, Changsha 410008, China; baiyongping@csu.edu.cn

**Keywords:** heart maturation, gene network, alternative splicing, hub gens, OgdhL

## Abstract

Heart maturation is an essentially biological process for neonatal heart transition to adult heart, thus illustrating the mechanism of heart maturation may be helpful to explore postnatal heart development and cardiac cardiomyopathy. This study combined proteomic analysis based on isobaric tags for relative and absolute quantitation (iTRAQ) and transcriptome analysis based on RNA sequencing to detect the proteins and genes associated with heart maturation in mice. The proteogenomics integrating analysis identified 254 genes/proteins as commonly differentially expressed between neonatal and adult hearts. Functional and pathway analysis demonstrated that these identified genes/proteins contribute to heart maturation mainly by regulating mRNA processing and energy metabolism. Genome-wide alternative splicing (AS) analysis showed that some important sarcomere and energy-associated genes undergo different AS events. Through the Cytoscape plug-in CytoHubba, a total of 23 hub genes were found and further confirmed by RT-qPCR. Next, we verified that the most up-regulated hub gene, *Ogdhl*, plays an essential role in heart maturation by detecting energy metabolism phenotype changes in the Ogdhl-interfering cardiomyocytes. Together, we revealed a complex gene network, AS genes and patterns, and candidate hub genes controlling heart maturation by proteome and transcriptome combination analysis.

## 1. Introduction

The vertebrate heart is the first organ to form during the process of embryogenesis [1]. In mice, perinatal cardiac growth forms the four ventricular chambers, but the fetal heart cannot efficiently afford the hemodynamic and body growth demands after birth [2]. To meet the rapid growth, the fetal heart needs to undergo maturation to develop into an adult heart; whereby a small, with less efficient contraction, hyperplasic, and glycolytic fetal heart becomes a large, with robust contraction, hypertrophic, and mitochondrial oxidative phosphorylation adult heart [3,4,5,6,7]. The fetal–adult heart maturation mainly includes four aspects, structural maturation, functional maturation, cell-cycle maturation, and metabolism maturation. Previous experiments have demonstrated that these four maturation aspects interacted with each other, and changing one aspect often alters other aspects [8]. For example, structural maturation increases myofibril alignment promoting contractile force and functional maturation. Then functional maturation requires the heart to use energy more efficiently, driving energy maturation acceleration. Structure and functional maturation limit cardiomyocyte depolymerization, enhancing difficulty for cell division and promoting cell cycle maturation. Likewise, metabolism maturation often improves the sarcomeric structure and inhibits the cell cycle, leading structure and cell cycle maturation. Cell cycle maturation also accelerated sarcomere alignments and structure maturation, leading energy maturation. Therefore, the heart maturation processes might interact in co-regulated circuits, and a common regulatory mechanism may exist to control heart maturation.

High-throughput information by bioinformatics analysis is a powerful tool to explore heart maturation regulatory mechanisms. Previous transcriptome profiles have extensively sketched the differentially expressed genes and functional pathways for heart maturation [9,10,11,12,13]. However, protein is the direct performer of cellular biological function, which means that heart proteomics may provide additional assistance to interpret the heart maturation mechanism. Thus, we seek to combine transcriptomic and proteomic analysis to obtain a more comprehensive and reliable characterization of heart maturation.

In this study, we integrated proteogenomics analysis to explore the genes and proteins regulating heart maturation. A total of 5432 genes and mapped 847 proteins were differentially expressed between neonatal and adult hearts. Venn analysis demonstrated that 254 genes were common changed in proteomic and transcriptomic levels and well correlated by Pearson’s correlation test. Functional annotation indicated that these 254 genes were mainly enriched in energy metabolism and mRNA processing. Alternative splice (AS) analysis was performed, and some sarcomere and energy-associated genes such as *Myh7*, *Mpc1*, and *Coq8a* were found to undergo different AS events. Next, hub genes were identified by Cytoscape plug-in CytoHubba and validated by RT-qPCR, including nine hub genes (*Rps8*, *Rps13*, *Rpl8*, *Rpl7a*, *Rpl3*, *Rpl13a*, *Rpl10a*, *Rps16*, and *Rpl13*) in neonatal and 14 hub genes (*Scp2*, *Ogdhl*, *Hadhb*, *Fabp3*, *Etfdh*, *Ech1*, *Cpt2*, *Acadvl*, *Acadm*, *Acad11*, *Slc25a20*, *Decr1*, *Acsl1*, and *Acox1*) in adult heart. The most up-regulated (≥10 fold) hub gene *Ogdhl*, as a core enzyme of the 2-oxoglutarate dehydrogenase multienzyme complex, was confirmed to regulate energy metabolism maturation in cardiomyocytes. Together, our study characterized the gene expression changes, regulatory networks, alternative splicing, and hub genes in neonatal and adult hearts.

## 2. Materials and Methods

### 2.1. Animals and Heart Tissue Preparation

The adult hearts were harvested in C57BL/6N male mice (postnatal 60 days) and the neonatal hearts were obtained from the same strain mice while on the day of birth (postnatal 0 day). Anesthetized by 5% isoflurane, each mouse was fixed and the chest was opened to expose the heart. Then, the heart was quickly excised and the excess of fatty tissue or blood vessels was removed by micro-ophthalmic scissors. The hearts were then immediately freeze-clamped in liquid nitrogen and stored at −80 °C refrigerator. Every group contained at least three mouse hearts, and all heart samples were processed after collection as soon as possible. The animal protocols were approved by the Institutional Animal Care and Use Committee (IACUC), Fuwai Hospital, Chinese Academy of Medical Sciences (Approval number: Fw-2018-009).

### 2.2. Protein Extraction, Peptide Digestion, and Analysis by LC-MS/MS

For protein extraction, heart tissue was extracted by the SDT (4% (*w*/*v*) SDS, 100 mM Tris/HCl pH7.6, 0.1 M DTT) method and quantified by BCA assay. The model number of LC-MS/MS (Q Exactive Orbitrap LCMSMS, thermo fish, Waltham, MA, USA).Filter-aided sample preparation (FASP) method was performed to proteolysis the heart sample by trypsin digest, and the peptides were desalted using a C18 cartridge. Next, the resulting peptides were lyophilized and re-suspended in 40 µL of 0.1% formic acid solution and quantification (OD280). Each sample was separately performed by the HPLC liquid system Easy nLC with a nanoliter flow rate. Buffer A solution was 0.1% formic acid aqueous solution, buffer B solution was 0.1% formic acid acetonitrile aqueous solution (acetonitrile is 84%). The chromatographic column was balanced with 95% A solution, and the autosampler loaded the sample onto the loading column (Thermo scientific acclaim pepmap100, 100 μm × 2 cm, nano Viper C18) and passed through the analytical column (Thermo scientific EASY column, 10 cm, ID 75 μm, 3 μm, C8-A2) for separation, and the flow rate was 300 nL/min. After chromatographic separation, the samples were analyzed by a Q-Exactive mass spectrometer. The detection method was the positive ion, the scanning range of precursor ion was 300–1800 *m*/*z*, the resolution of the primary mass spectrum was 70,000 at 200 *m*/*z*, the AGC (Automatica gain control) target was 1 × 10^6^, the maximum IT was 50 ms, and the dynamic elimination time was (Dynamic exclusion) 60.0 s. The mass-to-charge ratio of peptides and peptide fragments was collected according to the following method: 20 fragment maps (MS2 scan) were collected after each scan (full scan), MS2 activation type was HCD, Isolation window was 2 *m*/*z*, the resolution of the secondary mass spectrum was 17,500 at 200 *m*/*z*, the normalized collision energy was 30 eV, and the underfill is 0.1%.

### 2.3. Protein Identification and Quantification Based on iTRAQ Data

Proteins were identified and quantified by the software MaxQuant (version number 1.5.3.17). The relevant parameters are as follows: max missed cleavages: 2; fixed modifications: carbamidomethyl (C); variable modifications: oxidation (M); main search: 6 ppm; first search: 20 ppm; MS/MS tolerance: 20 ppm; database: uniprot_mouse_84433; database pattern: reverse; include contaminants: true; protein FDR ≤ 0.01; peptide FDR ≤ 0.01; peptides used for protein quantification: razor and unique peptides were used; time window (match between runs): 2 min; protein quantification: LFQ; and minimum ratio count: 1.

### 2.4. RNA Extraction, Library Preparation, and Transcriptome Sequencing

The heart RNA was extracted using the Trizol method (Invitrogen) as previously reported [14]. The amount and integrity of RNA were evaluated by the RNA Nano 6000 Assay Kit of the Bioanalyzer 2100 system (Agilent Technologies, Santa Clara, CA, USA). A total of 3 µg RNA per heart sample was employed as input material for RNA sample preparation. Briefly, the mRNA was extracted from 3 µg RNA using magnetic beads linked to poly-T oligonucleotides and then fragmented with divalent cations in fragmentation buffer. First-strand cDNA was synthesized from fragmented mRNA using random oligonucleotide as primer and with M-MuLV reverse transcriptase. Then, mRNA was degraded by RNaseH, and subsequently second-strand cDNA was synthesized using DNA polymerase I and dNTP. The purified double-stranded cDNA underwent end-repair, added A-tailing, and was connected to the sequencing adapter. Next, we screened 370~420 bp cDNA with AMPure XP beads, performed PCR amplification, re-purified the PCR products by AMPure XP beads, and finally obtained the library. After the library construction, the library was preliminarily quantified by Qubit2.0 Fluorometer and diluted to 1.5 ng/µL, and Agilent 2100 bioanalyzer was used to detect the insert size of the library, and sequencing was performed with the Illumina NovaSeq 6000 sequencer with 150 bp paired-end reads.

### 2.5. Processing of Sequence Data and Mapping Reads to Reference Genome

The raw data were processed by CASAVA base calling analysis, which converted image data to sequence data. To obtain clean data, reads with adapters, reads with base information not determined, and reads with low-quality (base number of Qphred ≤ 20 accounts for more than 50% of the entire read length) were excluded. Clean reads were mapped to the reference GRCm38 mouse genome sequence using HISAT2 (v2.0.5). The counts of genes were calculated by Feature Counts (1.5.0-p3), and DESeq2 software (1.20.0) was used to make comparison between different groups. Benjamini and Hochberg’s methods were used to adjust the obtained *p*-value (padj) to control the false discovery rate. Genes with padj ≤ 0.05 and |log2 (fold change)| ≥ 1 were defined as significant differential expression genes.

### 2.6. Functional Enrichment Analysis of Differentially Expressed Proteins and Differentially Expressed Genes

The functional pathway of differentially expressed genes (DEGs) in (Supplement Table S1) and differentially expressed proteins (DEPs) (Supplement Table S2) were analyzed by Metascape (https://metascape.org/gp/index.html (accessed on 23 October 2021)) [15], KOBAS2.0 website (http://kobas.cbi.pku.edu.cn/ (accessed on 2 October 2021)), or Proteomaps (http://bionic-vis.biologie.uni-greifswald.de/ (accessed on 19 October 2021)) [16,17].

### 2.7. Alternative Splicing Genes Analysis

Alternative splicing (AS) is an important mechanism for gene expression and protein regulation. The R language package rMATS (4.1.0) was used to analyze AS events, including five optional splicing events: skipped exon (SE), retained intron (RI), alternative 5′ splice site (A5SS), alternative 3′ splice site (A3SS), and mutually exclusive exon (MXE).

### 2.8. Hub Genes Analysis

The gene regulatory network (GRN) is composed of nodes (genes) and edges (the biological relationship). Hub genes were the top genes that showed a high correlation with other genes in GRN and play an important role in regulation of gene network. Referencing to previous studies [18], we constructed a protein–protein interaction network by String analysis (https://www.string-db.org/ (accessed on 10 November 2021)). Then used the Cytoscape plugin CytoHubba to identify hub genes in the neonatal and adult network. The top 20 hub genes were obtained by each algorithm and overlapped by upset analysis in the UpSetR R package.

### 2.9. Quantitative Real-Time PCR Analysis

The total RNA of neonatal and adult heart tissues was reverse transcript into cDNA following the manufacturer’s instructions (Takara Bio, Kusatsu, Japan). The real-time quantitative PCR determined the gene expression as previous reported [19], and the primers were listed in Supplementary Table S3. The 2^−ΔΔCt^ method was used to calculate the relative gene expression levels.

### 2.10. Primary Cardiomyocytes Mitochondrial Stress Assay

The primary cardiomyocytes were extracted from neonatal mice heart by the Neonatal Heart Dissociation Kit’s (Miltenyi Bio-Tech, Teterow, Germany) following the manufacturer’s instruction. Small interference RNAs (siRNAs) were obtained from Shanghai Heng Yuan Biological Technology, and the *Ogdhl* siRNA sequence was 5′- CCAGCTGTTCTCCAAGAAA-3′. The siRNA interference was conducted as described previously [20]. After 48 h interference by siRAN, the energy usage phenotype test assay was performed on cardiomyocytes by XF24 Extracellular Flux Analyzer (Seahorse Bioscience, Agilent, Santa Clara, CA, USA) following the manufacturer’s instruction.

## 3. Results

### 3.1. Proteomic Profiling of Neonatal and Adult Heart

**Figure 1:**
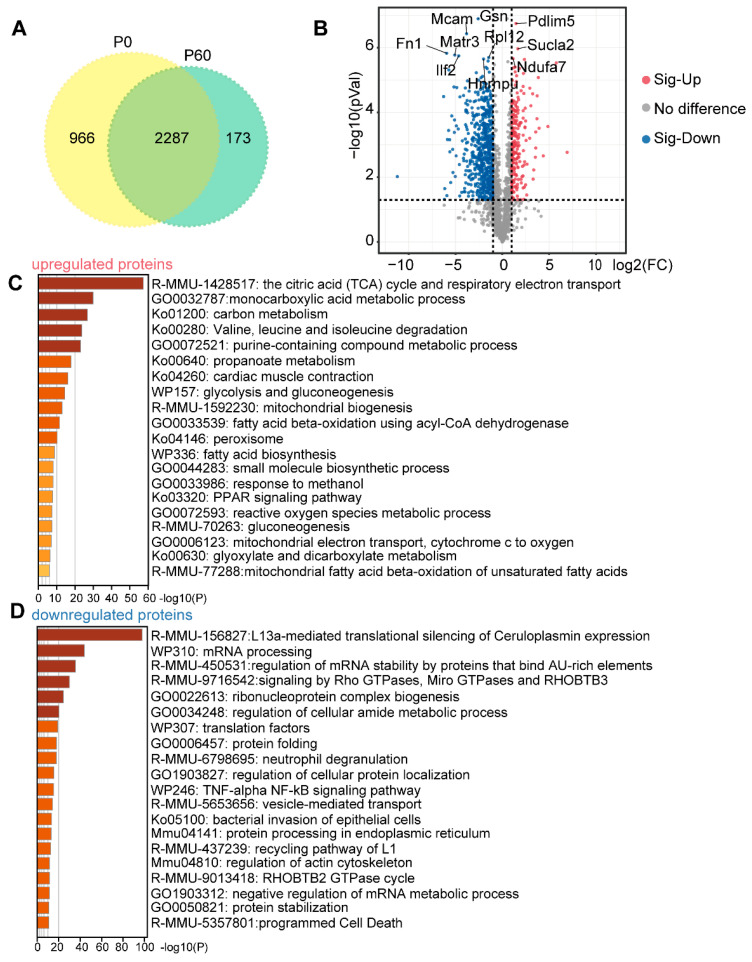
Proteome analysis of neonatal and adult hearts: (**A**) Venn analysis showed that there are 966 proteins specifically detected in the P0 heart and 173 in the P60 heart. While 2287 proteins are commonly expressed between the P0 and P60 heart. (**B**) Volcano analysis demonstrates that a total 847 proteins are defined as differentially expressed proteins (DEPs) between the P0 and P60 hearts, based on the threshold (padj ≤ 0.05 and |log2 (fold change)| ≥1), of which 626 are down-regulated proteins and 221 are up-regulated proteins. The top 10 significantly changed proteins are pointed out. (**C**,**D**) Functional analysis of up-regulated proteins (**C**) and down-regulated proteins (**D**) by Metascape, and the top 20 items are listed.

A label-free global proteomic profiling was performed on hearts from postnatal 0 days (P0, on the day of birth) and postnatal 60 days (P60) of C57BL/6N mice (*n* = 3 mice/group). Accordingly, 34,236 unique peptides were generated, and 3667 proteins were identified. Among all these detected proteins, 2287 proteins were commonly expressed in P0 and P60 hearts, while 966 proteins were specifically expressed in P0, and 173 proteins in P60 hearts (Figure 1A). Functional enrichment analysis using Metascape tools (https://metascape.org/ (accessed on 20 January 2022)) showed that strongly enriched categories for P0 heart were the regulation of mRNA metabolic process (GO1903311), ribonucleoprotein complex assembly (GO0022618), translation (GO0006412), and mitotic cell-cycle process (GO1903047) (Supplementary Figure S1A). In comparison, terms associated with P60 hearts were positive regulation of stress fiber assembly (GO0051496), sarcomere organization (GO0045214), NADH oxidation (GO0006116), hexose metabolic process (GO0019318), monocarboxylic acid metabolic process (GO0032787), and monocarboxylic acid metabolic process (GO0032787) (Supplementary Figure S1B). Next, we identified 847 differentially expressed proteins (DEPs), including 221 increased DEPs and 626 decreased DEPs between P0 and P60 (padj ≤ 0.05 and |log2 (fold change)| ≥ 1) (Figure 1B). Functional annotation revealed that tricarboxylic acid (TCA) cycle respiratory electron transport (R-MMU-1428517), cardiac muscle contraction (ko04260), fatty acid beta-oxidation using acyl-CoA dehydrogenase (GO0033539), and PPAR signalling pathway (ko03320) were enriched in P60-up-regulated DEPs (Figure 1C). Moreover, several processes of mRNA processing (WP310), Rho GTPases, Miro GTPases and RHOBTB3 signalling (R-MMU-9716542), and protein stabilization (GO0050821) were significantly relevant to P60 down-regulated DEPs (Figure 1D). In summary, these data reveal a profile that is associated with neonatal and adult heart protein levels during development.

### 3.2. Transcriptomic Profiling of Neonatal and Adult Heart

**Figure 2:**
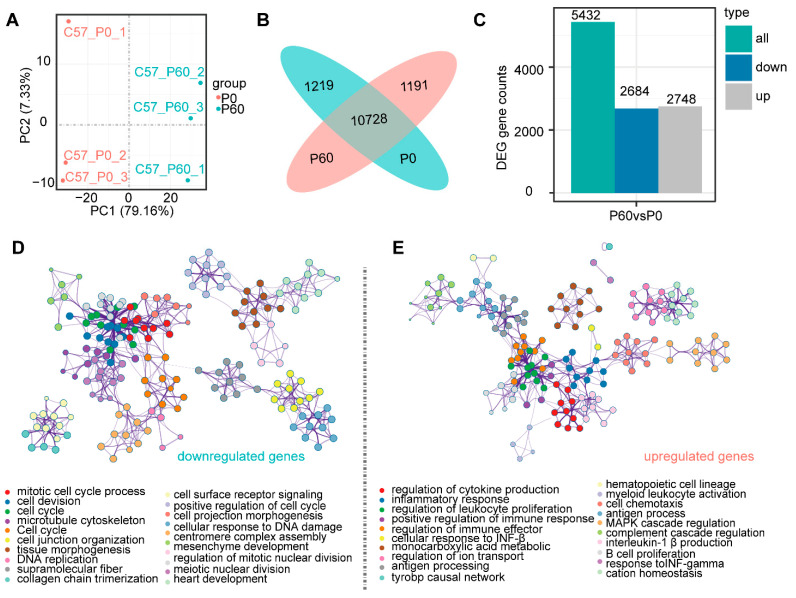
Transcriptome analysis of neonatal and adult hearts: (**A**) PCA analysis demonstrates that the neonatal and adult hearts belong to two distinct clusters. (**B**) Venn analysis shows the number of specificity and overlap genes among the neonatal and adult heart. (**C**) Differentially expressed genes (DEGs) between the neonatal and adult heart are presented, which contain a total 5432 DEGs following the threshold (padj ≤ 0.05 and |log2 (fold change)| ≥ 1) with 2684 down-regulated DEGs and 2748 up-regulated DEGs. (**D**,**E**) Functional signalling pathway of down-regulated DEGs (**D**) and up-regulated DEGs (**E**) by Metascape.

To verify transcriptional-level changes, we performed RNA-sequence analyses of the P0 and P60 hearts from an independent set of mice (*n* = 3 mice/group) (Figure 2A). The Illumina paired-end sequencing generated 44.91 and 45.44 million raw sequence reads, removing the adaptor and low-quality reads, we obtained 42.64 and 43.39 million clean reads for P0 and P60, respectively. The percentage of unique reads aligning with the genome sequence ranged from 48.15% for P0 to 44.46% for P60. Ultimately, 11,920 and 11,947 genes were identified in the P0 and P60 hearts (Figure 2A). Among these genes, 10,728 genes were commonly expressed in P0 and P60, while 1219 unique genes for P0 and 1192 genes for P60 were respectively identified (Figure 2B). Functional annotation showed that the P0 heart was associated with the mitotic cell-cycle process (GO1903047) and microtubule cytoskeleton organization (GO0000226) (Supplementary Figure S1C), while inflammatory response (GO0006954) and MAPK cascade (GO0000165) were enriched in the P60 heart (Supplementary Figure S1D). Compared to the P0 heart, the P60 heart had 5432 significant differentially expressed genes (DEGs), including 2748 up-regulated and 2684 down-regulated genes (padj ≤ 0.05 and|log2 (fold change) | ≥ 1) (Figure 2C). The following items: mitotic cell-cycle process (GO1903047) and extracellular matrix organization (GO0030198) were enriched in down-regulated DEGs (Figure 2D). While the up-regulated DEGs were annotated as being involved in the regulation of cytokine production (GO0001817), monocarboxylic acid metabolic process (GO0032787), and regulation of MAPK cascade (GO0043408) (Figure 2E). Together, these findings provide a gross profile of neonatal and adult hearts at transcriptome levels.

### 3.3. Compare Neonatal and Adult Heart by Proteomic and Transcriptome Combined Analysis

**Figure 3:**
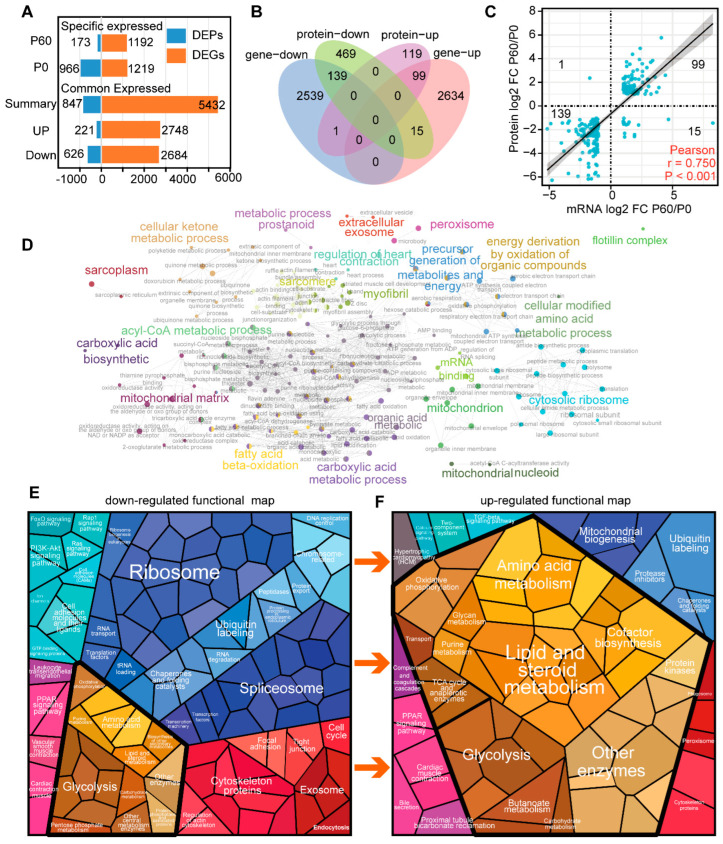
Proteogenomics integrating analysis of neonatal and adult heart: (**A**) The differentially expressed genes (DEGs) and differentially expressed proteins (DEPs) identified in neonatal and adult heart in different comparison. (**B**) Venn diagram shows the common expressed mRNAs/proteins in the neonatal and adult heart. (**C**) Pearson analysis demonstrates that the common mRNA/protein was well relative. (**D**) Cyto-scape plugin CytoGO shows the complex network of common changed mRNA/protein for the neonatal and adult heart. (**E**,**F**) The Proteomap shows the proportion change in down-regulated (**E**) and up-regulated (**F**) functional models.

To obtain a deep comparison between P0 and P60 hearts, we performed a combined analysis of proteomic and transcriptomic profiles (Figure 3A). Using integrated mRNA/protein data, we identified 254 genes that significantly change at both the proteomic and transcriptomic levels (Figure 3B). Almost all these genes (238 mRNA/protein) demonstrated a positive correlation between mRNA and protein expression by Pearson’s correlation test (*r* = 0.75, *n* = 254, *p* < 0.001), with 139 genes enriched in the P0 heart and 99 genes enriched in the P60 heart (Figure 3C). A hierarchical ontology tree of these 254 mRNA/proteins was constructed by Cytoscape plug-in ClueGo, where each node represented an enriched term, and the majority of top gene ontology (GO)terms were related to mRNA processing, energy metabolism, and heart structure development (Figure 3D).

Pathway analysis demonstrated that 139 down-regulated mRNAs/proteins were associated with cytoplasmic ribosomal proteins (WP163), mRNA processing (WP310), and translation initiation complex formation (R-MMU-72649) in the P0 heart (Supplementary Figure S1E). In contrast, up-regulated 99 mRNAs/proteins were involved in the monocarboxylic acid metabolic process (GO0032787), acyl-CoA metabolic process (GO0006637), PPAR signalling pathway (ko03320), and regulation of cardiac muscle contraction by calcium ion signalling (GO0010882) in the P60 heart (Supplementary Figure S1F). Proteomap analysis intuitively showed that the proportion of ribosome and energy metabolism modules dramatically changed between neonatal and adult hearts, suggesting that ribosome activity and energy maturation may be the major dominant aspects of heart maturation (Figure 3E).

The Venn diagram shows 27 mRNAs/proteins were overlapped in P0 and 94 mRNAs/proteins in the P60 heart, respectively, meaning that these genes were specifically expressed in neonatal and adult at transcriptomic and proteomic levels. These specific genes/proteins in P0 were associated with degradation of the extracellular matrix (R-MMU-1474228) and negative regulation of hydrolase activity (GO0051346) (Supplementary Figure S1G), while were enriched in sarcomere organization (GO0045214) in the P60 heart (Supplementary Figure S1H). Only a small group of genes (16 of 254) showed opposite expression trends at mRNA and protein levels, including a substantial number of structure genes (*Itgb6*, *Tmod4*, *Calr*, *Nexn*, and *Ktn1*) that up-regulated at the transcriptional level but down-regulated at the protein level in the P60 heart. Together, these finding indicated that the mRNA processes by ribosome and energy metabolism were significantly associated with heart maturation by proteogenomics integration analysis.

### 3.4. Alternative Splicing Mediating mRNA Process in Neonatal and Adult Heart

**Figure 4:**
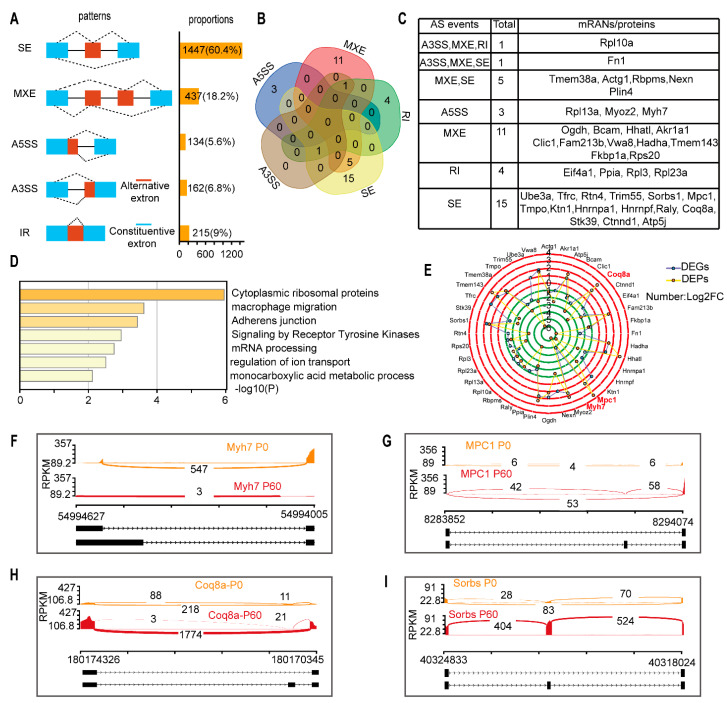
Alternative RNA processing changes occur during heart maturation: (**A**) Alternative splicing (AS) events categorized by different patterns and the proportion of total AS events in neonatal and adult heart. SE: skipped exon, MXE: mutually exclusive exon, A3SS: alternative 3′ splice acceptor site, A5SS: alternative 5′ splice acceptor site, IR: intron retained. (**B**,**C**) Forty AS genes overlapped with differentiated expressed mRNAs/proteins and Venn analysis demonstrates the AS events distribution (**B**) and the detailed information are listed (**C**). (**D**) Functional analysis of these 40 AS genes by Metascape. (**E**) AS gene expression change at transcript and protein levels. (**F**,**H**), RNA sequencing read of *Myh7* undergoes A5SS (**F**), *Mpc1* (**G**), *Coq8a* (**H**), and *Sorbs* (**I**) undergoes SE in the P0 and P60 hearts.

Since mRNA processing was dominant functional pathway, we decided to analyze the alternative splicing (AS) between neonatal and adult hearts. Since, AS is a primary transcription and post-transcriptional regulation mechanism for mRNA, leading to two or more mRNAs generated from the same precursor mRNA (pre-mRNA) by different splice sites. A total of 1658 genes underwent 2395 AS events, including 162 (6.8%) alternative 3′ splice acceptor site, 134 (5.6%) alternative 5′ splice acceptor site, 437 (18.2%) mutually exclusive exon, 215 (9%) retained intron, and 1447 (60.4%) skipped exon, following the threshold false discovery rate (FDR) < 0.05 (Figure 4A). Moreover, almost all these AS genes (1647/1658) were DEGs (padj ≤ 0.05 and |log2 (fold change)| ≥ 1).

To explore whether AS regulates gene expression in neonatal and adult hearts, we overlapped AS genes with 254 common mRNAs/proteins described above and found that a subset of 40 mRNAs/proteins underwent 49 AS events (Figure 4B). The distribution of AS patterns are as follows: 15 genes exon skipped, 11 genes mutually exclusive exon, four genes retained intron, three genes alternative 5’ splice site, and seven genes underwent two or more AS events (Figure 4C). Notably, these AS genes were associated with adherens junction (ko04520) and monocarboxylic acid metabolic process (GO0032787) (Figure 4D), which were the main terms for heart structure and metabolism. Moreover, AS genes exhibited a least a one fold discrepancy between transcript and protein levels (Figure 4E), resulting in a protein expression change often more dramatic than transcription. For instance, *myosin heavy polypeptide 7* (*Myh7*), which encodes heart contraction molecular motor proteins, was alternatively spliced at individual 5’ splice sites, generating an exon deficiency transcript that decreased severely at the protein level in the adult heart (Figure 4F). Energy-associated genes, *mitochondrial pyruvate carrier 1* (*Mpc1*) (Figure 4G), *coenzyme* Q8A (*Coq8a*) (Figure 4H), and *sorbin* and *SH3 domain containing 1* (*Sorbs1*) (Figure 4I) underwent exon skipping, activating an exonic splicing enhancer and increasing protein expression levels in the adult heart. Collectively, these results indicate that sarcomere and energy-associated gene expression changes between the neonatal and adult heart, which may be determined by alternative splicing during maturation.

### 3.5. Network Analysis for hub Genes in Neonatal and Adult Heart

**Figure 5:**
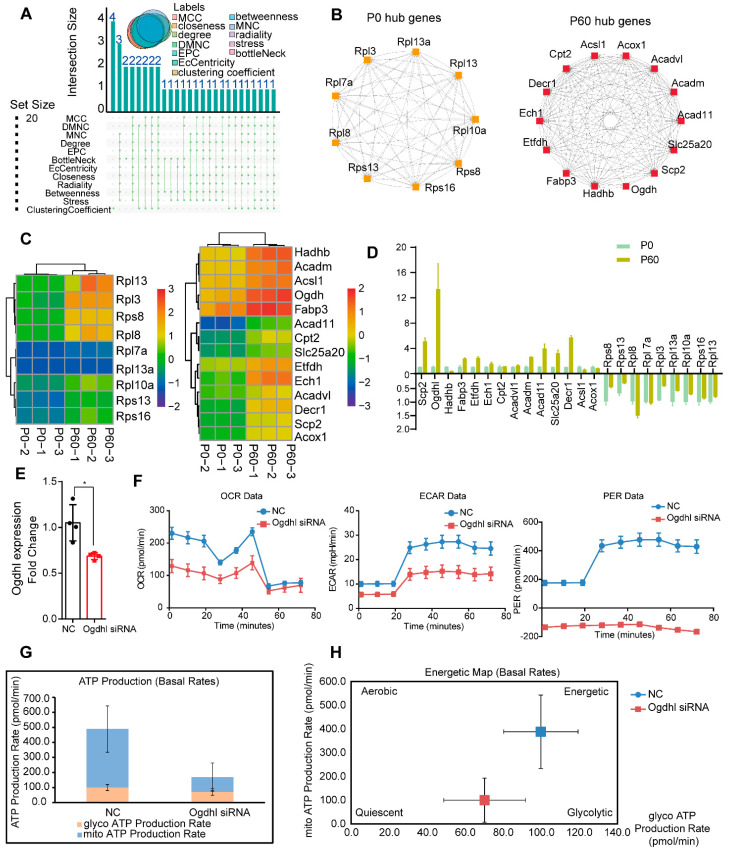
Hub genes analysis during heart maturation: (**A**) Upset analysis demonstrates hub genes analyzed in Cytoscape plug-in Cytohubba by different algorithms. (**B**) P0 and P60 hub genes are exhibited. (**C**) Heatmap analysis shows gene expression change in hub genes in the P0 and P60 heart. (**D**) qRT-PCR validates hub genes expression in P0 and P60 heart. (**E**) qRT-PCR found that the expression of *Ogdhl* was down-regulated almost 40%. * *p* < 0.05. (**F**) ATP energy production by primary cardiomyocyte upon NC or *Ogdhl* siRNA interference, OCR (oxygen consumption rate), ECAR (extracellular acidification rate), and PER (per efficiency rate). (**G**) ATP production rate of NC and *Ogdhl* siRNA interference cardiomyocytes is demonstrated. (**H**) And the energy map shows the Ogdhl cardiomyocyte is more quiescent.

Given the profound molecular discrepancy in the P0 and P60 hearts, we decided to find the hub genes that exhibit significant correlations with other genes in the neonatal and adult hearts. We separately constructed networks for these two stages based on the 139 mRNAs/proteins in the P0 and 99 mRNAs/proteins in the P60 heart. By Search Tool for the Retrieval of Interacting Genes (STRING) analysis, and visualized in Cytoscape, we obtained 128 nodes/1682 protein-interaction pairs for the P0 and 86 nodes/710 protein-interaction pairs for the P60 heart. The top 20 hub genes were defined by the Cytoscape plug-in CytoHubba based on the following algorithms, including maximal clique centrality (MCC), the density of maximum neighborhood component (DMNC), maximum neighborhood component (MNC), edge percolated component (EPC), EcCentricity, bottleneck, degree, closeness, radiality, betweenness, stress, and clustering co-efficient. Upset analysis was used to exhibit the hub genes distribution by each algorithm, and hub genes of at least eight algorithms that overlapped were selected, containing nine hub genes (*Rps8*, *Rps13*, *Rpl8*, *Rpl7a*, *Rpl3*, *Rpl13a*, *Rpl10a*, *Rps16*, and *Rpl13*) in the P0, and 14 hub genes (*Scp2*, *Ogdh*, *Hadhb*, *Fabp3*, *Etfdh*, *Ech1*, *Cpt2*, *Acadvl*, *Acadm*, *Acad11*, *Slc25a20*, *Decr1*, *Acsl1*, and *Acox1*) in the P60 heart (Figure 5A,B). Proteomap analysis was used to characterize hub genes’ function and status, and it showed that down-regulated hub genes *Rps8*, *Rps13*, *Rpl8*, *Rpl7a*, *Rpl3*, *Rpl13a*, *Rpl10a*, *Rps16,* and *Rpl13* were all enriched in the ribosome (Figure 3E and Supplementary Figure S2A). In comparison, up-regulated hub genes were rate-limiting enzymes in fatty acid metabolism. For instance, *Scp2*, *Decr1*, and *Etfdh* being divided into enzymes; *Slc25a20* into fatty acid transport process; *Hadhb*, *Cpt2*, Acadm, and *Acadvl* into lipid and steroid metabolism; *Ogdh1* into TCA cycle; and *Ech1* into the peroxisome (Figure 3F and Supplementary Figure S2B). Taken together, these results provide potential genes that may be closely associated with ribosome activity and energy metabolism during heart maturation.

### 3.6. The Ogdhl Gene Play Essential Role in Cardiomyocyte Energy Metabolism

To further verify the role of these hub genes in heart development, hub gene expression levels were demonstrated by heatmap (Figure 5C). RT-qPCR was used to confirm expression change in the P0 and P60 hearts, and the most significant change gene was *Ogdhl*, whose expression changes increased almost 10-fold in the P60 heart (Figure 5D). OGDHL is a core subunit of the 2-oxoglutarate dehydrogenase multienzyme complex, and is involved in the tricarboxylic acid cycle and energy metabolism. The siRNA oligonucleotides were used to interfere Ogdhl expression and the knockdown efficiency was almost 40%, as confirmed by qRT-PCR (Figure 5E). ATP synthesis and ATP content can indicate the function of mitochondrial energy metabolism; thus, the energy phenotype test by Seahorse XF24 extracellular-flux analyzer was performed. The oxygen consumption rate (OCR) and extracellular acidification rate (ECAR), an estimate of mitochondrial respiration and glycolysis, were measured to uncover how *Ogdhl* influences cardiomyocytes energy production and utilization. The ATP production analysis test showed that impaired mitochondrial respiration, glycolysis, and efficiency rate in *Ogdhl* deleted primary cardiomyocytes (Figure 5F), and the metabolic state become quiescent with *Ogdhl* low expression (Figure 5G,H). Taken together, these results demonstrate a preliminary validation of the functions of the hub gene *Ogdhl* in heart maturation.

## 4. Discussion

The murine heart is an electrically driven pump, sustained and rhythmic beating throughout life that guarantees adequate nutrient and oxygen supply to all tissues and organs of the body. Thus, as the body grows, the need to pump blood increases, requiring the heart to undergo essential maturation processes. Maturation is vital to establish proper heart functions in adults; defective heart maturation often exacerbates cardiomyopathy and even heart failure [21]. Moreover, in-clinic cell therapy using human embryonic or induced pluripotent stem cell-derived cardiomyocytes is limited because they lack immaturity [22,23,24]. Hence, understanding the precise mechanisms and critical molecular underlying heart maturation is of great importance toward heart development, cardiomyopathy, and cell therapy.

In this study, we integrated transcriptome and proteomics analysis to identify differentially expressed genes/proteins, functional pathways, alternative splicing, and hub genes from neonatal to adult heart development. Differentially expressed genes/proteins were subdivided into two classes: the specific genes/proteins expressed in neonatal or adult hearts, and the other being the common genes but differentially expressed between these two stages. Specific genes/proteins enriched in neonatal were simultaneously associated with the ribosome and mRNA process. While there was a little distinguish between the proteome and transcriptome in the adult heart, the top rank items related to inflammatory response were at the transcriptome but not the proteome. Previous studies also demonstrated the enrichment of immune-related pathways in the adult heart by RNA sequencing [11]. We considered that no noticeable expression change in inflammation proteins might address why adult hearts did not suffer unnecessary inflammatory interference with so many immune gene changes. Similar to the specific genes function in the neonatal and adult heart, the up-regulated common genes/proteins in adults were enriched in energy metabolism and sarcomere structure while being associated with mRNA processing and ribosome activity in down-regulated genes/proteins, suggesting that mRNA processing and metabolism may be the most critical determinants for heart maturation.

Splicing is the process of removing non-coding regions and joining all coding regions together to edit precursor mRNA into mature mRNA. Alternative splicing of mRNA transcripts refers to selectively including or excluding different parts of the pre-mRNA to form different transcript isoforms, and is the main factor affecting protein diversity, especially during development. Previous studies have reported that AS transitions play a decisive role in heart development, which titin (*Ttn*) is regulated by muscle-specific splicing factor RBM20 [25]. Moreover, alternative splicing has been confirmed to mediate isoform transitions, such as troponin T type 2 (*Tnnt2*), calcium/calmodulin-dependent protein kinase 2D (*Camk2d*), and anaphase-promoting complex subunit 11 (*Anapc11*) in the human fetal and adult heart [26,27]. Our study found that several sarcomere and energy metabolism associated genes, *Myh7*, *Atp5j*, *Mpc1*, and *Coq8a*, undergo different AS events during neonatal and adult heart transition, and AS-altered expression changed more dramatic at protein levels than transcriptome levels. Furthermore, it has been reported that RNA processing, such as splicing and transportation, has been shown to regulate proliferation and the cell cycle, and RNA processing enhancing may be a consequence of the cell cycle, suggesting that mRNA processing in the neonatal heart probably is associated with proliferation.

The mRNA processing genes were dominant in neonatal heart and energy metabolism genes in the adult heart, indicating that hub genes in these two stages may be distinguished. Thus, we explored hub genes in these two stages separately. Cytoscape plug-in CytoHubba was chosen referring to previous studies, and different hub genes were obtained based on different algorithms, so we selected hub genes where at least eight algorithms overlapped to improve accuracy. In neonatal heart, eight hub genes (*Rps8*, *Rps13*, *Rpl8*, *Rpl7a*, *Rpl3*, *Rpl13a*, *Rpl10a*, *Rps16*, and *Rpl13*) were all related to the ribosome, suggesting that ribosome activity was a potential influence factor for neonatal heart function. Fourteen hub genes (*Scp2*, *Ogdhl*, *Hadhb*, *Fabp3*, *Etfdh*, *Ech1*, *Cpt2*, *Acadvl*, *Acadm*, *Acad11*, *Slc25a20*, *Decr1*, *Acsl1*, and *Acox1*) in the adult heart were enriched in energy metabolism. By gene annotations, we found that these hub genes were revolved around the critical steps about fatty acid oxidation, including fatty acid uptake, carnitine cycle, β-oxidation cycle, acyl-CoA dehydrogenases, and the TCA cycle. For instance, the *Fabp3* mainly transported long-chain fatty acids and their acyl-CoA esters intracellularly [28]. The *Cpt2* and *Slc25a20* encoded carnitine palmitoyltransferases and carnitine acylcarnitine translocase, control acylcarnitines transport into mitochondrial matrix for beta-oxidation [29]. The *Acox1*, as the first enzyme of the β-oxidation cycle, catalyzed the desaturation of acyl-CoAs to 2-trans-enoyl-CoAs; and the *Acadvl*, *Acadm*, and *Acad11*, belonging to very long-chain acyl-CoA dehydrogenase, medium-chain acyl-CoA dehydrogenase, and acyl-CoA dehydrogenase 11 separately, degraded different chain length-specific fatty acids [30]. The *Hadhb* encoded mitochondrial trifunctional protein beta subunit harbors enoyl-CoA hydratase (S)-3-hydroxy acyl-CoA dehydrogenase 3-ketothiolase activities, which are specific for long-chain intermediates [31]. The *Ogdhl*, 2-oxoglutarate dehydrogenase (E1-like) component of the 2-oxoglutarate dehydrogenase multienzyme complex, mediates the decarboxylation of alpha-ketoglutarate in the TCA cycle, and we validated that *Ogdhl* expression was the most dramatic change between neonatal and adult heart [32]. The *Ogdhl* silencing of cardiomyocytes found that energy metabolism decreased, suggesting that *Ogdhl* may be the critical regulator for heart energy maturation.

Nevertheless, a more in-depth heart maturation mechanism is still required because the actual biological function of hub genes or AS genes lacks systemic and genetic biological assays. As part of future research, we intend to verify the function of these hub genes for heart maturation. Furthermore, future investigations at various maturation time points and deeper sequencing approaches, including peptidomics, metabolomics, and single-cell sequencing, would be needed to detect more precise heart maturation mechanisms.

## 5. Conclusions

In conclusion, combined with proteomic and transcriptomic analysis, we described the profile of differential expression genes, functional pathway, alternative splicing, and hub genes in the neonatal and adult heart. We found that mRNA processing and energy metabolism were the two dominant aspects that control the maturation process. Neonatal hub genes were mainly associated with the ribosome, suggesting that ribosome activity may potentially regulate heart proliferation. Almost all adult hub genes were enzymes controlling critical steps of fatty acid oxidation, and we primitively verified hub gene *Ogdhl* in cardiomyocyte energy metabolism.

## Data Availability

The data and methods that are included in this study are available from the corresponding author upon reasonable request. Sequencing data are deposited in the National Center of Biotechnology Information (accession number PRJNA783509), and BioSample database (SAMN23435408, SAMN23435409, SAMN23435410, SAMN23435411, SAMN23435412, SAMN23435413).

## Supplementary Materials

The following supporting information can be downloaded at: https://www.mdpi.com/article/10.3390/genes13020250/s1, Figure S1: Functional analysis of neonatal and adult hearts, Figure S2: Hub genes during heart maturation, Table S1: The differentially expressed genes between neonatal and adult heart, Table S2: The differentially expressed proteins between neonatal and adult heart. Table S3: The primer sequence of hub genes.
