# Supplementary material for: Proteogenomics Integrating Reveal a Complex Network, Alternative Splicing, Hub Genes Regulating Heart Maturation
Source: Genes (Basel). 2022 Jan 28;13(2):250. doi: 10.3390/genes13020250 (PMC8872128; doi:10.3390/genes13020250)
Supplement: Supplementary file 1 [file genes-13-00250-s001.zip › genes-1511139-supplementary/Supplement Figure 1-2.pdf]

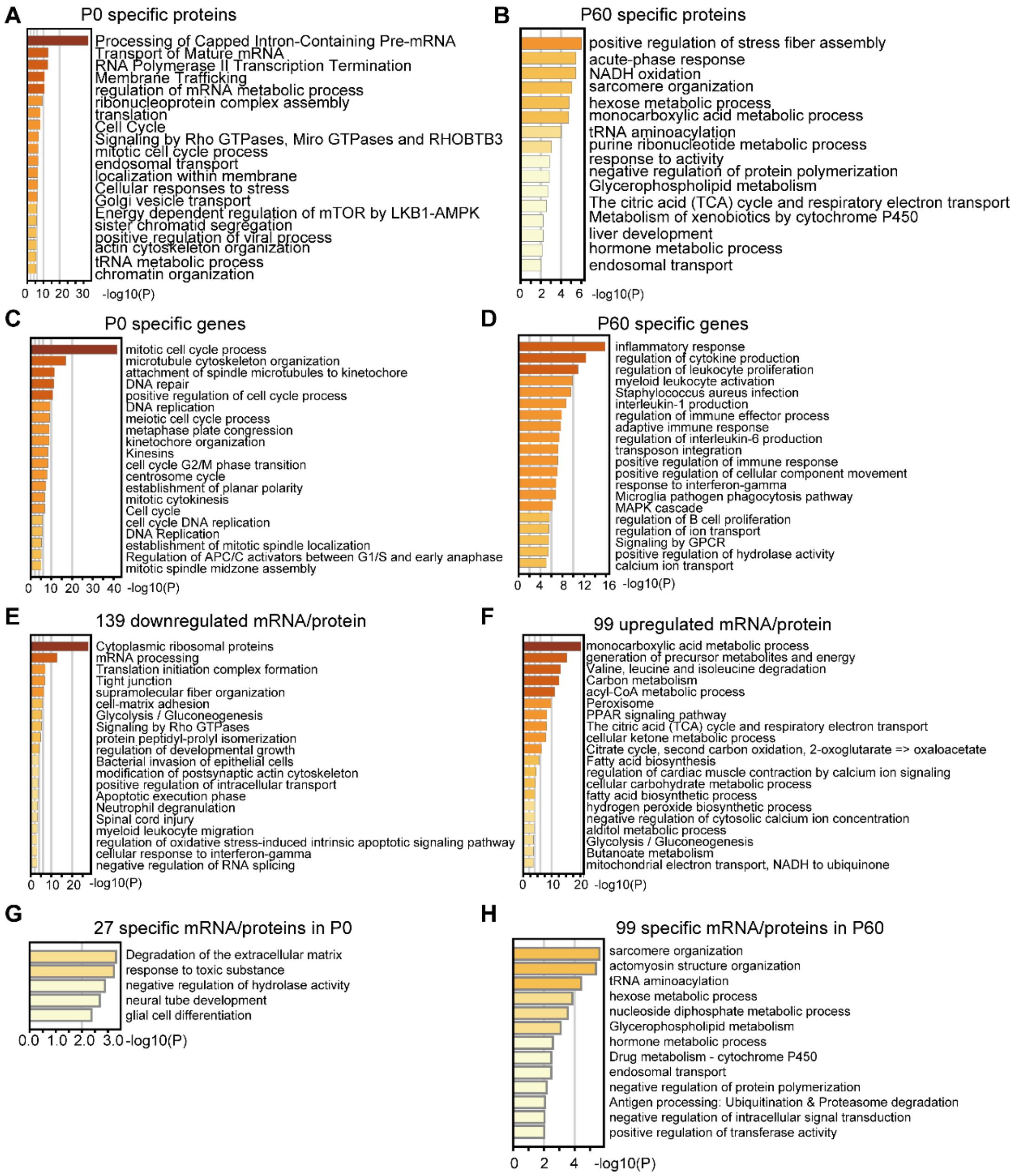

Figure S1. Functional analysis of neonatal and adult hearts. A-B, Functional analysis of specific proteins in P0 (A) and P60 (B) heart by metascape analysis. C-D, Pathways of specific genes in P0 (C) and P60 (D) heart by metascape analysis. E-F, Detail terms associated with common expressed mRNA/proteins, and downregulated (E) and upregulated (F) mRNA/proteins enriched terms were separately demonstrated. G-H, Specific mRNA/proteins enriched in P0 (G) and P60 (H) heart were exhibited.

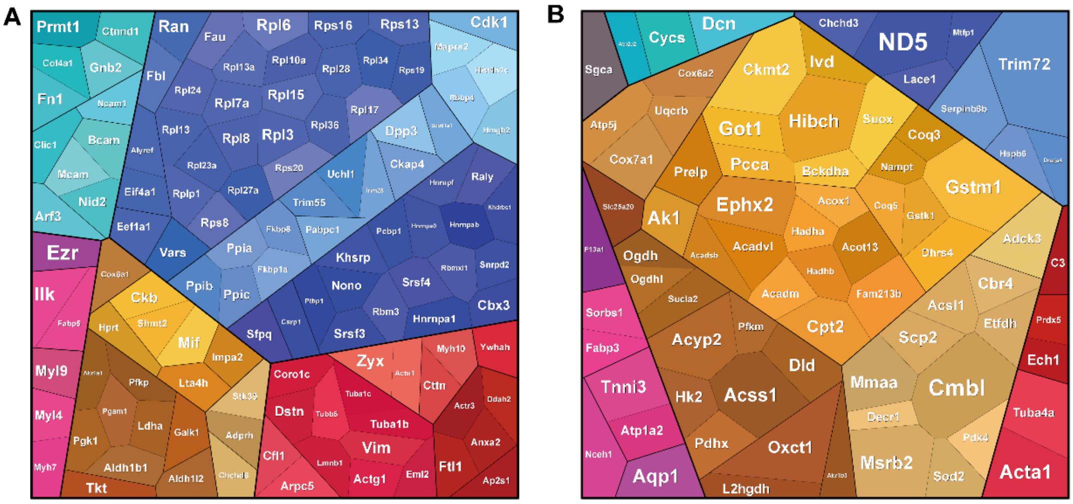

Figure S2. Hub genes during heart maturation. A-B, Protein map demonstrated central genes in every functional mode in P0 (E) and P60 (F) heart.
